# The Medical News Visiting List for 1902

**Published:** 1902-01

**Authors:** 


The Medical News Visiting List for 1902. Weekly (dated for 30 patients); Monthly (undated, for 120 patients per month); Perpetual (undated, for 30 patients weekly per year); and Perpetual (undated, for 60 patients weekly per year). The first three styles contain 32 pages of data and 160 pages of blanks. The 60-patient Perpetual consists of 256 pages of blanks. Each style in one wallet-shaped book, with pocket, pencil and rubber. Seal grain leather, $1.25. Thumb-letter index, 25 cents extra. Philadelphia and New York: Lea Brothers & Co., Publishers.

